# Modeling the Importance of Within- and Between-County Effects in an Ecological Study of the Association Between Social Capital and Mental Distress

**DOI:** 10.5888/pcd16.180491

**Published:** 2019-06-13

**Authors:** Tse-Chuan Yang, Stephen A. Matthews, Feinuo Sun, Marina Armendariz

**Affiliations:** 1 Department of Sociology, University at Albany, SUNY, Albany, New York; 2 Department of Sociology and Criminology, Pennsylvania State University, University Park, Pennsylvania; 3 Department of Biobehavioral Health, Pennsylvania State University, University Park, Pennsylvania

## Abstract

**Introduction:**

Levels of mental distress in the United States are a health policy concern. The association between social capital and mental distress is well documented, but evidence comes primarily from individual-level studies. Our objective was to examine this association at the county level with advanced spatial econometric methods and to explore the importance of between-county effects.

**Methods:**

We used County Health Rankings and Roadmaps data for 3,106 counties of the contiguous United States. We used spatial Durbin modeling to assess the direct (within a county) and indirect (between neighboring counties) effects of social capital on mental distress. We also examined the spatial spillover effects from neighboring counties based on higher-order spatial weights matrices.

**Results:**

Counties with the highest prevalence of mental distress were found in regional clusters where levels of social capital were low, including the Black Belt, central/southern Appalachia, on the Mississippi River, and around some Indian Reservations. Most of the association between social capital and mental distress was indirect, from the neighboring counties, although significant direct effects showed the within-county association. Models also confirmed the importance of county-level socioeconomic status.

**Conclusion:**

We found that county social capital is negatively related to mental distress. Counties are not isolated places and are often part of wider labor and housing markets, so understanding spatial dependencies is important in addressing population-level mental distress.

**Summary**

**What is already known on this topic?**

At the individual level, high levels of social capital are associated with low levels of mental distress.

**What is added by this report?**

We used ecological data to demonstrate that social capital and prevalence of mental distress are spatially clustered in US counties. We showed that social capital decreases the prevalence of mental distress within a county, but this within-county association is weaker than the between-county association.

**What are the implications for public health practice?**

Our results suggest that policy interventions to promote population-level mental health should consider broader multi-county contexts and the coordination of actions within the consortia of neighboring counties.

## Introduction

Mental distress refers to a range of symptoms and experiences that cause problems in the way individuals think, feel, or behave (1). Levels of mental stress in the United States have increased since the 1990s (2). Social capital reflects the potential resources, both tangible and invisible, embedded in social relations or networks. Social capital can be measured at both individual and ecological levels (3), and its association with mental distress has been well documented (4–6). However, most evidence comes from individual-level studies, and the ecological findings are inconclusive due to diverse analytic units and methods used (4,6).

We identified 2 major gaps in our understanding of how social capital is associated with mental distress at the ecological level. First, research overlooks the spatial dependencies between these 2 measures by simplifying the contribution of neighboring areas into a single parameter estimate, which is also sometimes misinterpreted (7). Second, whether mental distress of a focal area is affected by the social capital of neighboring areas remains underexplored, and little is known about the importance of distance — as measured by spatial order (ie, *n^th^* spatial lags) — in explaining spillover effects of social capital on mental distress.

To address these gaps, we applied spatial Durbin modeling approaches to the County Health Rankings and Roadmaps (CHRR) data set (8) for the United States (9). We examined 2 hypotheses: 1) within a county, higher levels of social capital are associated with lower prevalence of mental distress, even after controlling for other confounders; and 2) the levels of mental distress of a county are negatively influenced by the low social capital of neighboring counties, and the effect is strongest from the neighboring adjacent counties than from counties farther away.

## Methods

### Data and measures

The 2018 CHRR synthesizes both health and socioeconomic information from national data sets, such as the Behavioral Risk Factor Surveillance System (BRFSS), the Dartmouth Atlas of Health Care, and the American Community Survey. Although the 2018 CHRR covers all US counties, we focused on the counties in the contiguous United States (N = 3,106). All data were publicly available, so no institutional review board approval was needed.

The dependent variable was county-level mental distress. Mental health considers stress, depression, and problems with emotions, and this measure emphasizes those residents with more chronic and severe mental health issues (8). In the BRFSS it was measured with “frequent mental distress,” which is the percentage of adults who reported more than 14 days in response to the question, “How many days during the past 30 days was your mental health not good?” For the counties with limited data, the entire BRFSS sample and census population estimates were used to estimate this variable (8).

The key independent variable was the social capital index developed by Rupasingha and colleagues (10), which was created by applying principal component analysis (PCA) to 4 variables: number of establishments per 10,000 population, voter turnout, census response rate, and number of nonprofit organizations. Higher social capital index values refer to stronger social connections among residents. This social capital index has been used in county level analysis (11,12), but its application to mental health is limited.

We also considered other covariates. The socioeconomic status (SES) index is a PCA-derived score using percentage of population older than 25 who have at least some college education (factor loading = 0.805), unemployment rate (factor loading = −0.752), child poverty rate (factor loading = −0.935), and logged median household income (factor loading = 0.900). Approximately 72% of variation among these 4 variables can be captured with a single factor; a higher SES index score indicates higher socioeconomic status.

Several variables reflect the demographic composition of a county. Age was measured with percentage of population younger than 18 and percentage of population older than 65. Racial/ethnic composition was based on percentage of non-Hispanic blacks, non-Hispanic Asians, and Hispanics. We included percentage population that was female, not proficient in English, and living in a rural area, and we included the ratio of household income at the 80th and 20th percentiles. We checked the variance inflation factors among the independent variables and found that all were smaller than 4, indicating that multicollinearity was not a concern.

### Analytic approach

To test our hypotheses, we used the spatial Durbin model, developed in spatial econometrics but rarely used in health research. A spatial Durbin model can be expressed as follows (7,13):

(*I_n_* – *ρW*)*y* = *αl_n_* + *Xβ* + *WXθ* + *ε*

where both the spatially lagged dependent (*ρWy*) and independent variables (*WXθ*) are included (9). The endogeneity in the model makes the interpretations of the estimates richer (7). Explicitly, the spatial Durbin model allows researchers to separate the direct (within a county) and indirect (to/from neighboring counties) effects of an independent variable on the dependent variable. The equation above can be rewritten:

*y* = (*I_n_* – *ρW*)^−1^ *αl_n_* + (*I_n_* – *ρW*)^ −1^ *Xβ* + (*I_n_* – *ρW*)^−1^ *WXθ* + (*I_n_* – *ρW*)^ −1^ *ε*

The partial derivatives of *y* with respect to the *r*th independent variable (*X_r_*) across the *n* observations in the study region can be expressed as follows:

*∂y*/*∂X_r_* = (*I_n_* – *ρW*)^ −1^(*I_n_β_r_* + *Wθ_r_*)

where *∂y*/*∂X_r_* indicates an *n *x* n* matrix, and *β_r_* and *θ_r_* represent the parameter estimates associated with the independent variable in a county and in neighboring counties. Several implications from this equation highlight the benefit of a spatial Durbin approach (7,13,14). Specific to this study, the third equation indicates that the change in a county’s social capital index will not only lead to the change in mental distress in the same county, but also influence the frequent mental distress in other counties. The former refers to the direct effects [average of the main diagonal elements of (*I_n_* – *ρW*)^ −1^(*I_n_β_r_* + *Wθ_r_*) matrix], whereas the latter indicates the indirect effects (average of the off-diagonal elements). Furthermore, the partial derivatives of *y* are a function of (*I_n_* – *ρW*)^ −1^ and can be expanded as a linear combination of powers of the spatial weights matrix (*W*): *I_n_ + ρW + ρ^2^W^2^ + ρ^3^W^3^ +* … . The powers of *W* correspond to the counties themselves (zero-order), adjacent neighbors (first-order), neighbors of adjacent neighbors (second-order), and so on. It is possible to partition both the direct and indirect impacts of social capital on mental distress by using the powers of spatial weight matrix. Consequently, researchers can generate a “spatial profile” of the importance of neighboring areas with the partitioning results. In this way we can test the second hypothesis.

Although other forms of spatial econometrics models handle spatial association (eg, spatial lag and spatial error), the spatial Durbin model is the most appropriate spatial regression form, particularly when the generating process underlying the observed data is unknown (7). Both spatial and aspatial exploratory data analysis were done before the spatial Durbin and partitioning analysis. The Markov chain Monte Carlo method was used to calculate the direct and indirect effects and the partitioning results. All analyses were conducted with the *spdep* package (15) in R (16). Comparisons between the spatial Durbin model and other conventional spatial models are available on request.

## Results

On average, 12% of the adult population aged 18 to 85 in a county reported more than 14 days of mental distress in the past 30 days (Table 1). The relatively small standard deviation of mental distress (1.88) suggests that in most counties at least 8.5% of adults aged 18–85 reported mental distress. The social capital index had a mean value of 0 and a standard deviation of 1.26. 

**Table 1:** Descriptive Statistics of Variables (N = 3,106), Ecological Study of the Association Between Social Capital and Mental Distress, County Health Rankings and Roadmaps, United States, 2018

Variable	Minimum	Maximum	Mean (Standard Deviation)
Adults reporting mental distress, %	8.03	21.34	12.21 (1.88)
Social capital index	−3.18	21.81	0.00 (1.26)
Socioeconomic status index	−3.80	2.99	0.00 (1.00)
Younger than 18 y, %	5.15	40.79	22.34 (3.40)
Older than 65 y, %	4.63	56.31	18.45 (4.51)
Non-Hispanic black, %	0.00	85.15	9.03 (14.37)
Non-Hispanic Asian, %	0.00	36.50	1.40 (2.41)
Hispanic, %	0.50	96.25	9.33 (13.73)
Not English proficient, %	0.00	32.69	1.75 (2.93)
Female, %	27.80	56.55	49.93 (2.22)
Rural resident, %	0.00	100.00	58.52 (31.44)
80th/20th income ratio	0.00	8.93	4.52 (0.74)

Counties with high prevalence of mental distress (4th and 5th quintiles) were concentrated in the South (particularly the Black Belt), central and southern Appalachia, and the Mississippi River Valley through Oklahoma (Figure). Clusters of high levels of mental distress were also found in Indian Reservations (eg, the Four Corners and several counties in the Dakotas). 

**Figure:**
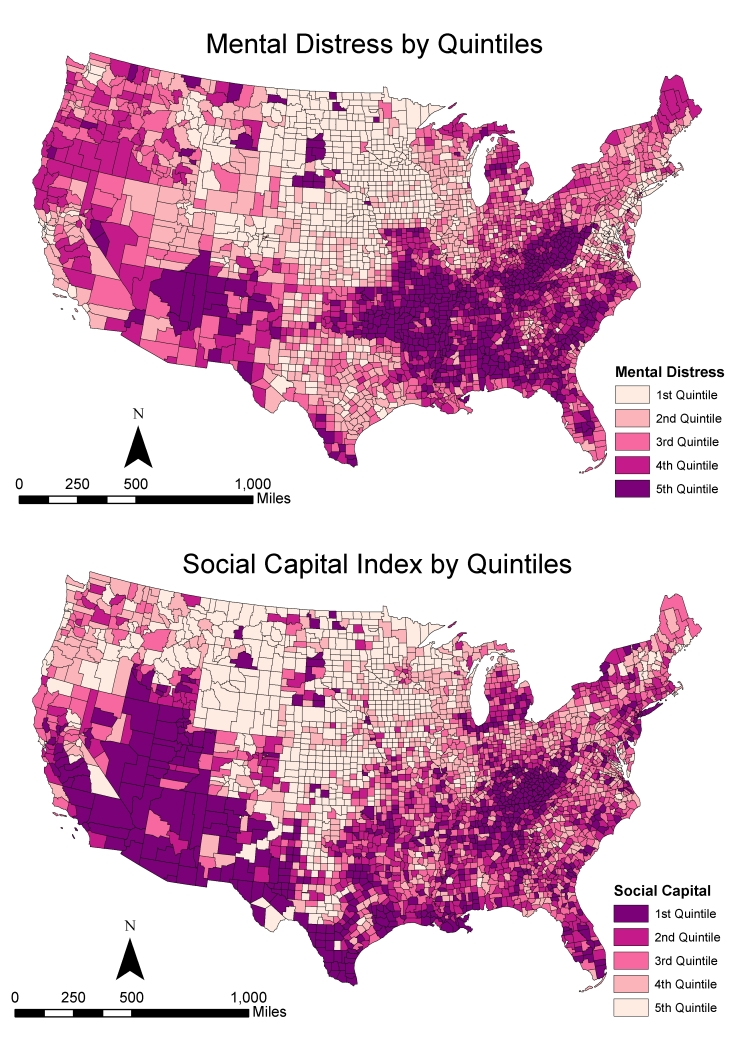
Spatial distribution of mental distress and social capital in the United States, County Health Rankings and Roadmaps, United States, 2018. Map A depicts county-level prevalence of mental distress, and Map B depicts the social capital index of counties.

The spatial distribution of social capital was the opposite of the spatial distribution of mental distress. Many counties with high prevalence of mental distress had low social capital. Counties with high social capital and low mental distress were clustered in the Great Plains and the Midwest. Overall, spatial analyses indicated that mental distress and social capital are negatively associated. 

The spatial Durbin modeling results are shown in Table 2. The direct effect of social capital (−0.087) was smaller than the indirect effect. A one-unit increase in the average social capital index in neighboring counties was associated with a 0.234 percentage point decrease in mental distress of a focal county. The indirect effect of social capital on mental distress was roughly 2.7 times (−0.234 divided by −0.087 = 2.69) stronger than the direct effect.

**Table 2:** Decomposition Estimates of the Direct and Indirect Effects on Percentage of Adults Reporting Mental Distress, Ecological Study of the Association Between Social Capital and Mental Distress, County Health Rankings and Roadmaps, United States, 2018

Variable	Direct Effect	Indirect Effect	Total
Social capital index	−0.087^a^	−0.234^a^	−0.321^a^
Socioeconomic status index	−1.263^a^	−0.532^a^	−1.795^a^
Younger than 18 y, %	0.018^a^	−0.102^a^	−0.084^a^
Older than 65 y, %	−0.075^a^	0.005	−0.070^a^
Non-Hispanic black, %	−0.010^a^	−0.009	−0.020^a^
Non-Hispanic Asian, %	−0.014	0.009	−0.004
Hispanic, %	−0.028^a^	0.005	−0.023^a^
Not English proficient, %	0.006	−0.010	−0.004
Female, %	0.114^a^	0.132^a^	0.247^a^
Rural resident, %	0.001	−0.002	−0.001
80th/20th income ratio	0.315^a^	−0.472^a^	−0.158

a Significant at *P* < .05.

SES index had the strongest total effect on mental distress (Table 2). A one-unit increase in SES index was associated with a 1.263 percentage point decrease in the prevalence of mental distress within a county. High SES in neighboring counties was associated with decreased mental distress (percentage point decrease, 0.532). The percentage of female population was associated with spatial variation in mental distress. For every one percentage point increase in female population, mental distress increased by 0.114. Furthermore, a higher income ratio (ie, higher income inequality) was associated with a higher mental distress level within a county; however, the indirect effect of income ratio was negatively associated with mental distress (−0.472). 

Results of how the effect of social capital and other covariates on mental distress are transited through neighboring counties are presented in Table 3. The direct effect of social capital at the zero-order (W_0_) was −0.072, indicating that almost 83% (−0.072 divided by −0.087 = 0.828) of the direct effect came from a county itself and the other 17% could be attributed to the inter-county dependencies. The immediate neighbors (W_1_) appeared not to matter, but the second-order neighboring counties contributed to the direct effect of social capital. The contribution of the third-order (W_3_) neighbors became much smaller, yet remained significant.

**Table 3:** Spatial Partitioning Results of Direct and Indirect Effects on Percentage of Adults Reporting Mental Distress, Ecological Study of the Association Between Social Capital and Mental Distress, County Health Rankings and Roadmaps, United States, 2018

Variable	Direct					Indirect				
	W_0_	W_1_	W_2_	W_3_	W_4_	W_0_	W_1_	W_2_	W_3_	W_4_
Social capital index	−0.072^a^	−0.002	−0.007^a^	−0.002^a^	−0.002^a^	−0.015	−0.062^a^	−0.039^a^	−0.032^a^	−0.023^a^
Socioeconomic status index	−1.229^a^	0.093^a^	−0.090^a^	−0.005^a^	−0.015^a^	0.742^a^	−0.448^a^	−0.168^a^	−0.183^a^	−0.122^a^
Younger than 18 y, %	0.024^a^	−0.006^a^	0.001	−0.001^a^	0.000	−0.047^a^	−0.011	−0.013^a^	−0.008^a^	−0.006^a^
Older than 65 y, %	−0.076^a^	0.007^a^	−0.005^a^	0.000	−0.001^a^	0.057^a^	−0.021^a^	−0.005	−0.007	−0.005^a^
Non-Hispanic black, %	−0.010^a^	0.001	−0.001^a^	0.000^a^	0.000^a^	0.004	−0.004^a^	−0.002^a^	−0.002	−0.001^a^
Non-Hispanic Asian, %	−0.014	0.002	−0.001	0.000	0.000	0.013	−0.002	0.000	−0.001	0.000
Hispanic, %	−0.028^a^	0.003^a^	−0.002^a^	0.000	0.000^a^	0.022^a^	−0.007^a^	−0.001	−0.002	−0.001^a^
Not English proficient, %	0.006	−0.001	0.000	0.000	0.000	−0.007	0.000	−0.001	0.000	0.000
Female, %	0.106^a^	−0.005^a^	0.008^a^	0.001^a^	0.002^a^	−0.039^a^	0.054^a^	0.027^a^	0.025	0.017^a^
Rural resident, %	0.001	0.000	0.000	0.000	0.000	−0.001	0.000	0.000	0.000	0.000
80th/20th income ratio	0.345^a^	−0.048^a^	0.020^a^	−0.004^a^	0.002^a^	−0.388^a^	0.017	−0.043^a^	−0.013	−0.014

a Significant at *P* < .05.

Estimates of indirect effect of social capital decreased from the first-order to the higher orders. More than 25% (−0.062 divided by −0.234 = 0.265) of the indirect effect came from the first-order neighbors, but higher-order neighbors still contributed to the overall indirect effect.

## Discussion

We found strong evidence for our first hypothesis, that county social capital is negatively related to mental distress and that this relationship holds even after considering other confounders. The direct effect of social capital on the prevalence of mental distress was negative and significant in the spatial Durbin model. The partitioning results further indicated that more than 80% of the direct effect was within-county and that neighboring counties strengthened the association of social capital with mental distress.

Our study is explicitly ecological, and the findings contribute to the wider literature of social capital and mental distress. How do we understand this relationship? On one hand, our social capital index reflects the potential connections and social ties among residents (3,10). These social relations create trust and reciprocity that can be used to cope with negative emotions, stress, anxiety, and depression (4,6,17). Individuals living in counties with strong social capital receive better social support than those living in areas with weak social capital. As a result, the prevalence of mental distress decreases with the increase in social capital. Additionally, strong social capital facilitates a community’s capacity for action and cooperative social activities (18,19), which produces an environment conducive to economic development and community well-being (20). Although the findings suggesting that social capital buffers against the potential negative impacts of economic and social adversities are inconclusive, our results support the ecological finding that social capital may lower population prevalence of mental distress.

We also hypothesized that the prevalence of mental distress of a county is negatively associated with social capital of neighboring counties and this relationship decreases with distance (ie, increasing spatial lag order). Our results support this hypothesis but also indicate that the spatial spillover effect is complex. The indirect effect of social capital on mental distress was roughly 2.7 times stronger than the direct effect, suggesting a strong spatial spillover effect from neighboring counties. This indicates that counties with low prevalence of mental distress benefit indirectly from the strong social capital of neighboring counties. Our results suggest that the first-order neighbors are the most important contributors. Although other neighbors remain connected, their contributions decline as spatial order increases. The spatial clustering patterns in the Figure highlighted the strong spatial dependence embedded in social capital and mental distress, and the partitioning results showed how the indirect effects work through spatial adjacency.

As the indirect effect is reciprocal, it indicates that a one-unit increase in social capital of a focal county was related to a 0.234 percentage point decrease in mental distress of neighboring counties. The significant indirect effect of social capital confirms that the spatial association between social capital and mental distress was not only a within-county phenomenon, but influenced by inter-county spatial dependencies, which is captured by the exogenous interactions ((*I_n_* – *ρW*)^ −1^ WX) in the model. The nonsignificant zero-order indirect effect of social capital (−0.015) suggests that the indirect effect of social capital may be largely due to spatial spillover associated with the spatial structure (ie, form of W) and the spillover effect from the first-order neighbors was more crucial than from neighboring counties at the higher orders. 

Our study has limitations. The results and conclusions may change if the underlying data are aggregated to different geographies, which is a modifiable area unit problem (21,22). As a sensitivity analysis, we included county area in the model and found that county size is not statistically related to mental distress. That is, while large counties may have neighbors that are geographically farther away than smaller counties, our findings are unaltered. Furthermore, our conclusions cannot be generalized to the individual level (23). There is no consensus on how to measure social capital at the aggregate level, and the social capital index may not fully reflect the complexities of this construct (3,24). Other mental health measures (eg, mental disorders) should be considered to bolster the beneficial effect of social capital on mental health at the county level. Importantly, both mental distress and social capital are not race/ethnicity specific, which limits our understanding of the dynamics between these 2 variables within each racial/ethnic group. This warrants future endeavors to develop race/ethnicity-specific measurements. Moreover, given the programming limitation, our analysis does not weigh the influence of a county on another by total population. Future research should incorporate population size into the spatial weight matrix. Finally, the analysis is cross-sectional, and it is possible that high prevalence of mental distress leads to weak social capital. The causal relationship between these 2 variables needs to be clarified.

The spatial dependencies between social capital and mental distress provide insights. First, the spatial spillover process generates an indirect effect (from neighboring counties) that is stronger than the direct effect (within a county). The spatial dependencies cannot be identified with conventional spatial regression models, which in part may explain why ecological-level evidence is mixed (4). Future ecological work should consider spatial dependencies to address some of the inconsistencies in the social capital literature. Second, related to policy implications, the indirect effect of social capital on mental distress suggests that improving social capital in a certain county will have spillover effects and reduce mental distress in adjacent or nearby counties. In addition, cross-county collaboration to improve social capital and connections (ie, regional interventions) should be considered to maximize the effect of social capital on mental health.

Beyond social capital, we found that SES played a critical role in explaining the prevalence of mental distress. SES has the strongest impact on mental distress within a county, and this indicates that the spatial variation in mental distress may be a consequence of spatial economic inequality (25). The importance of SES has been discussed (2,26,27), and our results confirm this relationship. Income ratio is also important and our finding echoes the literature, suggesting income inequality follows the social relativity theory as neighbors with high income inequality reduce the sense of relative deprivation, which in turn improves population health (28). Moreover, prevalence of mental distress increases with the percentage of female population within (and beyond) a county. This positive association also corresponds to the extant literature (2,29). It is plausible that females encounter unique social and psychological stressors (eg, pregnancy and social role expectations) that affect their mental distress (2,6,29), although men’s reluctance to disclose mental distress may also be a factor (30). The importance of these variables is confirmed in sensitivity analysis where all the independent variables are standardized (results available on request), indicating that social capital and SES are not only statistically significant but also play a substantive role in explaining the spatial variation in mental distress.

In sum, we contribute to the mental health literature in 2 ways. First, we provide robust evidence for the beneficial association between aggregate levels of social capital and mental distress in US counties. Second, our adoption of spatial Durbin models showed the complicated inter-county dependencies and the relationship between these 2 measures. To our knowledge, no prior research has used the spatial Durbin modeling to clarify how levels of mental distress in a county are affected by neighboring counties.
